# Helicobacter cinaedi Infections in Emergency Departments: A Descriptive Study

**DOI:** 10.7759/cureus.44650

**Published:** 2023-09-04

**Authors:** Kento Izuta, Yoshinori Matsuoka, Toshikazu Hasuike, Yasukazu Hijikata, Yusuke Kuwahara, Daisuke Mizu, Koichi Ariyoshi

**Affiliations:** 1 Department of Emergency Medicine, Kobe City Medical Center General Hospital, Kobe, JPN; 2 Department of Infectious Diseases, Kobe City Medical Center General Hospital, Kobe, JPN; 3 Department of Healthcare Epidemiology, Graduate School of Medicine and Public Health, Kyoto University, Kyoto, JPN; 4 Graduate School of Health Management, Keio University, Fujisawa, JPN

**Keywords:** bacteremia, infected aortic aneurysm, vertebral osteomyelitis, helicobacter cinaedi, community-acquired infections

## Abstract

Aim: *Helicobacter cinaedi*, a Gram-negative spiral bacterium, is a rare cause of bacteremia in humans. Unfortunately, little is known about *H. cinaedi* infections in emergency departments (EDs). We aimed to describe the clinical features of *H. cinaedi* infections in the ED.

Methods: We conducted a descriptive study at the ED of Kobe City General Hospital (KCGH) in Japan between November 2011 and December 2020. We included all ED patients with *H. cinaedi *infections. We retrospectively obtained the patient data from electronic medical records and described the patient characteristics, clinical course, and management of *H. cinaedi* infections.

Results: A total of 22 patients in the ED were diagnosed with *H. cinaedi* infections, and all of them were detected through blood cultures. The chief complaints were vague: fever (18/22, 81.8%), chills (10/22, 45.5%), and localized pain or tenderness (8/22, 36.4%). Patients with complicated cases were also reported in the ED; three patients had vertebral osteomyelitis, two had infected aortic aneurysms, and another two had infected cysts (renal cyst and pancreatic cyst with concomitant empyema). Tetracycline (minocycline) was primarily prescribed and administered intravenously in five of 15 (33.3%) and orally in nine of 20 (45.0%) patients. Only one (4.5%) patient required surgical interventions. None of the patients died in the hospital.

Conclusions: We reported the clinical features of *H. cinaedi* infections in the ED. Although some patients developed complicated infections, the prognosis was not poor under appropriate treatment, and most of them were successfully treated with antibiotics, primarily tetracycline.

## Introduction

*Helicobacter cinaedi*, a Gram-negative spiral bacterium, is a rare cause of bacteremia in humans [1-4]. However, this microorganism is receiving growing attention because increasing numbers of human cases have been reported recently. Nevertheless, the mechanism of infections, as well as clinical aspects, remain incompletely understood. *H. cinaedi* can invade the bloodstream from the gastrointestinal tract, and this bacterial translocation is regarded as the first step of pathogenesis [5]. After entering the bloodstream, *H. cinaedi* bacteremia sometimes develops secondary foci of infections, such as cellulitis and arthritis, due to an affinity of the bacteria for the skin and joint tissues [1].

In earlier reports of *H. cinaedi *infections, most cases were considered opportunistic or nosocomial infections, especially among homosexual men and immunocompromised hosts [6-9]. However, community-acquired *H. cinaedi* infections have recently been reported [10-13]. Furthermore, infections have been reported across all age groups, from newborns to older adults, and include cases of cellulitis, gastroenteritis, arthritis, vertebral osteomyelitis, infected aortic aneurysm, and neonatal meningitis [1]. Currently, emergency physicians may be unfamiliar with the diverse clinical presentations of *H. cinaedi* infections, and the clinical features of *H. cinaedi* in the emergency department (ED) are inadequately described. We aimed to provide further insights into the clinical features and management of *H. cinaedi* infections in ED settings.

## Materials and methods

Study design and settings

This descriptive study was conducted at the ED of Kobe City General Hospital (KCGH) in Japan from November 2011 to December 2020. KCGH is a tertiary care hospital with 760 beds and serves 1.5 million habitants in the city, including both urban and rural areas of approximately 554 km^2^. As a certified critical care center, the ED has an average of 35,000 patient visits and 10,000 ambulance calls per year. On average, half of the severely injured patients in the city are managed in this ED.

Emergency physicians attend to patients in the ED and determine whether they should be admitted. In the ED, we take culture specimens from specific organs based on chief complaints, physical examination, and diagnostic tests. Furthermore, we obtain blood cultures from the following indications: fever (>38°C), shivering chills, unexplained loss of consciousness, circulatory disturbance, an increased respiratory rate (>20 breaths per minute), metabolic acidosis, an elevated or markedly decreased inflammatory response such as white blood cell counts and C-reactive protein levels, or suspected infective endocarditis. If patients are discharged from the ED after blood cultures are obtained, they are contacted immediately when the blood cultures yield positive results.

Selection of participants

The target population comprised patients with culture-confirmed *H. cinaedi* infections who presented to the ED. First, we selected all patients with *H. cinaedi *infections identified in any type of specimen from the electronic microbiology database that contains all data of culture specimens in the hospital. We determined whether *H. cinaedi* was a causative pathogen or not mainly based on clinical diagnoses that the treating physicians determined at their final follow-up day. Infected sites were determined in the same way. In addition, two investigators (KI and YM) independently reviewed medical histories, physical assessments, diagnostic tests including imaging tests, and overall clinical courses in the electronic medical records. When there was a discrepancy between our decision and clinical diagnosis, we planned to inquire about diagnoses to treating physicians. Finally, we excluded patients who visited the general outpatient ward and those infected with *H. cinaedi* identified from culture specimens following hospitalization.

Data collection

We retrospectively obtained the following patient data from the electronic medical records: age, sex, comorbidities (diabetes mellitus, chronic kidney disease (serum creatinine concentration ≥ 2.0 mg/dL), any malignancy, diseases requiring immunotherapy (use of steroids or immunosuppressive drugs), malignant diseases requiring recent chemotherapy (within 30 days of the ED visit)), infected site, laboratory data (white blood cell counts and C-reactive protein levels) from the initial ED visit, incubation days of blood cultures, antibiotic susceptibility, method of antibiotic administration (intravenous and oral), durations of antibiotic therapy (days), in-hospital days, complications or surgical interventions, and in-hospital mortality.

Cultures for *H. cinaedi*

We collected the data of all patients with *H. cinaedi *infections from the database at the bacteriological laboratory, including the results of all cultures from any kind of specimen. Blood cultures were observed for at least seven days and processed at the center’s laboratory using the BACTEC FX system (Nippon Becton, Dickinson, and Company, Tokyo, Japan). Polymerase chain reaction (PCR) identification was performed in all cases where Gram-negative spiral bacilli were identified using Gram staining. The KAPA2G Robust PCR Kit (NIPPON Genetics Company, Tokyo, Japan) was used to perform the test. *H. cinaedi* was identified using *gyrB*-targeted PCR (195 bp; forward primer, AGGATTCCACAAAGTGAGC; reverse primer, TCTGTGTCCTGTGCGTTCATC). 

Statistical analysis

Continuous data were expressed as the median (with interquartile range (IQR)). Categorical data were expressed as counts and percentages/proportions. To describe the patient characteristics and clinical course of *H. cinaedi* more informatively for emergency physicians, the patients were stratified into those who were admitted at initial presentation to the ED and those who were discharged from the ED. These features were compared between the groups using the χ^2 ^test for categorical variables and the Mann-Whitney U test for continuous variables.

Ethical approval

This study was approved by the Ethics Committee of KCGH (approval number: zn21114). The current study was conducted in accordance with the guidelines of the Declaration of Helsinki for medical research involving human participants. Informed consent was disclosed through an opt-out consent process in accordance with the Ethical Guidelines for Medical and Health Research Involving Human Subjects in Japan.

This article was previously posted to the Research Square preprint server on June 9, 2022.

## Results

Descriptive statistics and clinical covariates

**Figure 1:**
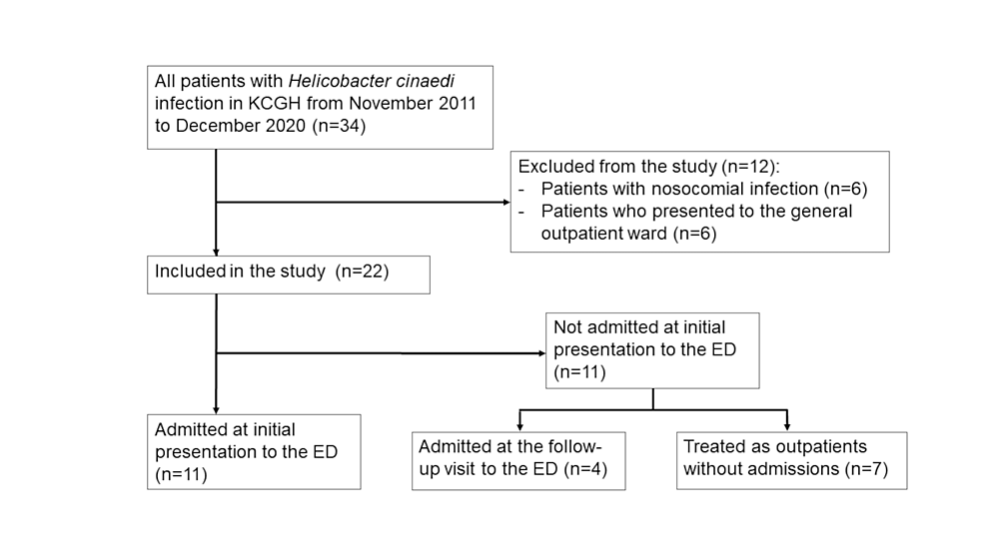
Patient flow of the study ED, emergency department; KCGH, Kobe City General Hospital

During the 10-year study period, a total of 22 ED patients were diagnosed with *H. cinaedi* infections, and all isolates were detected in the blood samples (Figure 1).

Of the 22 patients, 18 (81.8%) were male (Tables 1, 2).

**Table 1:** Characteristics and outcomes of patients with H. cinaedi bacteremia who were admitted and discharged at their initial emergency department visits IQR: interquartile range †Admitted at the follow-up visit to the ED (n=4)

	Total	Admitted at initial visit	Discharged at initial visit	P-value
	(n=22)	(n=11)	(n=11)	
Age, median (IQR)	65.5 (49.3–63.3)	66 (53.0–78.0)	64 (49.5–67.5)	0.74
Male, n (%)	18 (81.8)	10 (90.9)	8 (72.7)	0.27
Comorbidities				
- Diabetes mellitus, n (%)	7 (31.8)	2 (18.2)	5 (45.5)	0.17
- With complications, n (%)	2 (9.1)	1 (9.1)	1 (9.1)	-
- Hematological malignancies or solid tumours, n (%)	5 (22.7)	4 (36.4)	1 (9.1)	0.13
- Recent chemotherapy, n (%)	4 (18.2)	4 (36.4)	0 (0)	0.027
- Chronic renal disease, n (%)	4 (18.2)	3 (27.3)	1 (9.1)	0.27
- Immunosuppressant use, n (%)	1 (4.5)	0 (0.0)	1 (9.1)	0.31
- None, n (%)	3 (13.6)	1 (9.1)	2 (18.2)	0.53
Focus of infection				0.19
- Cellulitis, n (%)	3 (13.6)	2 (18.2)	1 (9.1)	
- Vertebral osteomyelitis, n (%)	3 (13.6)	2 (18.2)	1 (9.1)	
- Infected aortic aneurysm, n (%)	2 (9.1)	2 (18.2)	0 (0.0)	
- Colitis, n (%)	2 (9.1)	0 (0.0)	2 (18.2)	
- Infected cyst, n (%)	2 (9.1)	2 (18.2)	0 (0.0)	
- Lymphadenitis, n (%)	1 (4.5)	0 (0.0)	1 (9.1)	
- Unknown, n (%)	9 (40.9)	3 (27.3)	6 (54.5)	
Laboratory data				
- White blood cell count (10^9^/mL), median (IQR)	9.4 (7.8–15.7)	8.6 (7.8–15.0)	11.8 (8.3–17.5)	0.6
- C-reactive protein (mg/L), median (IQR)	3.41 (1.49–10.8)	10.7 (1.59–12.9)	2.00 (1.32–4.39)	0.071
In-hospital days, median (IQR)	25 (7.5–35.5)	26 (9.5–39.5)	10 (7.5–17.5) ^†^	0.27
Complications or surgical interventions, n (%)	1 (4.5)	1 (9.1)	0 (0.0)	0.31
In-hospital mortality, n (%)	0 (0.0)	0 (0.0)	0 (0.0)	-

**Table 2:** Characteristics of patients with H. cinaedi bacteremia in the emergency department

Patient ID	Age (years)	Sex	Admission at initial presentation to the ED	Admission at follow-up visit	Symptoms						Vital signs at ED visit							Quick SOFA score at ED	Vital sings on admission							Comorbidities							Site of infection	Laboratory data at initial visits		Definitive antibiotics	Duration of antibiotics (days)	Initial admission wards/services	Intensive care unit stay	Ventilator use	In-hospital mortality or surgical interventions
					Fever	Skin rash	Lumber pain	Nausea/vomiting	Arthralgia	Others	Glasgow Coma Scale	Systolic blood pressure, mmHg	Diastolic blood pressure, mmHg	Pulse rate, per minute	Respiratory rate, breaths per minute	Percutaneous oxygen saturation (room air), %	Body temperature, centigrade		Glasgow Coma Scale	Systolic blood pressure, mmHg	Diastolic blood pressure, mmHg	Pulse rate, per minute	Respiratory rate, breaths per minute	Percutaneous oxygen saturation (room air), %	Body temperature, centigrade	Diabetes mellitus	Diabetes with complications	Any malignancy	Chemotherapy	Chronic renal disease	Immunosuppressant use	None		White blood cell, 10^9^/mL	C-reactive protein, mg/L						
1	63	Male	+	-	+	-	+	-	+	-	E4V5M6	117	58	112	20	93	38.0	1	E4V5M6	110	55	104	16	96	38.1	+	+	-	-	+	-	-	Vertebral Osteomyelitis	21.3	13.6	Minocycline (iv)	56	High care unit	-	-	-
2	78	Male	+	-	-	-	+	+	-	-	E4V5M6	156	66	58	28	97	37.1	1	E4V5M6	146	70	62	20	98	37.0	-	-	-	-	+	-	-	Vertebral Osteomyelitis	7.7	11.3	Minocycline (iv)	159	High care unit	-	-	-
3	47	Female	-	+	-	-	-	-	-	-	E4V5M6	100	77	90	20	95	36.7	1	E4V5M6	112	81	94	16	94	36.5	-	-	-	-	-	-	-	Unknown	2.7	0.59	Minocycline (iv)	14	General ward	-	-	-
4	34	Male	+	-	+	-	-	-	+	+	E4V5M6	140	76	99	20	96	39.1	1	E4V5M6	135	78	85	24	96	38.7	-	-	-	-	-	-	+	Infected cyst	15.7	31.3	Minocycline (iv)	42	High care unit	-	-	-
5	50	Male	-	-	+	+	-	-	-	+	E4V5M6	130	59	84	18	96	37.8	0	E4V5M6	127	65	70	16	96	38.2	-	-	-	-	-	-	-	Lymphadenitis	21.5	2.00	Minocycline (po)	15	None	-	-	-
6	43	Male	+	-	+	-	-	-	-	+	E4V5M6	117	56	110	24	96	36.4	1	E4V5M6	110	72	102	24	96	36.0	-	-	-	-	-	-	-	Infected cyst	14.2	33.0	Sulbactam/Ampicillin (iv)	21	High care unit	-	-	-
7	43	Male	-	+	+	-	-	+	-	-	E4V5M6	180	80	68	16	97	37.3	0	E4V5M6	169	85	72	16	98	37.0	+	+	-	-	+	-	-	Unknown	4.3	0.17	Sulbactam/Ampicillin (iv)	28	General ward	-	-	-
8	32	Male	+	-	+	-	-	-	-	-	E4V5M6	110	60	100	16	98	38.0	0	E4V5M6	125	72	96	16	96	38.2	-	-	+	+	-	-	-	Unknown	4.6	1.59	Cefepime(iv)	27	High care unit	-	-	-
9	65	Female	+	-	+	-	-	-	+	-	E4V5M6	114	57	82	24	95	38.6	1	E4V5M6	120	55	84	20	95	38.0	-	-	+	+	-	-	-	Unknown	4.7	0.83	Doxycycline (po)	23	High care unit	-	-	-
10	66	Male	-	+	+	-	+	-	-	-	E4V5M6	126	60	86	16	98	36.6	0	E4V5M6	125	70	70	16	98	37.0	+	-	-	-	-	-	-	Vertebral Osteomyelitis	15.7	1.93	Ampicillin (iv)	84	General ward	-	-	-
11	91	Male	-	+	+	-	-	-	+	-	E4V5M6	122	62	74	16	98	36.8	0	E4V5M6	115	60	78	16	98	37.2	-	-	-	-	-	-	-	Unknown	7.4	1.28	Minocycline (iv)	15	General ward	-	-	-
12	90	Male	+	-	+	+	-	-	-	-	E4V5M6	130	80	80	20	99	38.7	1	E4V5M6	125	76	73	20	99	38.4	-	-	+	+	+	-	-	Cellulitis	9.6	1.46	Minocycline (po)	17	High care unit	-	-	-
13	57	Male	-	-	+	-	-	+	-	-	E4V5M6	140	110	128	24	98	38.9	1	E4V5M6	155	120	110	20	98	38.9	-	-	-	-	-	-	+	Colitis	19.3	4.58	Sulbactam/Ampicillin (iv)	15	None	-	-	-
14	64	Female	-	-	+	+	-	-	-	+	E4V5M6	120	80	106	22	92	37.7	1	E4V5M6	115	72	97	22	94	37.5	+	-	+	-	-	+	-	Cellulitis	11.8	9.77	Minocycline (po)	14	None	-	-	-
15	49	Male	-	-	+	-	-	-	-	-	E4V5M6	135	90	110	20	96	37.8	1	E4V5M6	138	82	92	20	98	37.9	-	-	-	-	-	-	-	Unknown	9.1	1.36	Ampicillin (po)	14	None	-	-	-
16	68	Female	-	-	+	-	-	-	-	+	E4V5M6	144	67	124	16	99	38.6	0	E4V5M6	159	75	116	16	96	37.2	-	-	-	-	-	-	+	Colitis	12.0	4.19	Levofloxacin (po)	14	None	-	-	-
17	78	Male	+	-	+	+	-	-	-	+	E4V5M6	111	74	96	22	96	37.6	1	E4V5M6	115	70	90	20	94	37.9	+	-	-	-	-	-	-	Cellulitis	8.6	1.59	Cefazolin (iv)	11	High care unit	-	-	-
18	66	Male	+	-	+	-	-	-	-	+	E4V5M6	123	68	116	16	92	38.0	0	E4V5M6	120	65	100	16	93	38.5	-	-	-	-	-	-	-	Infected aortic aneurysm	16.5	10.7	Cefotiam (iv)	481	Intensive care unit	+	+	+
19	78	Male	+	-	+	-	-	-	-	+	E4V5M6	102	62	92	20	96	38.0	1	E4V5M6	110	63	96	18	95	38.2	-	-	-	-	-	-	-	Infected aortic aneurysm	7.9	12.1	Cefazolin (iv)	57	High care unit	-	-	-
20	75	Male	-	-	-	-	-	-	-	-	E4V5M6	139	67	86	16	94	36.0	0	E4V5M6	134	68	72	16	96	36.5	+	-	-	-	-	-	-	Unknown	9.1	2.63	Levofloxacin (po)	6	None	-	-	-
21	67	Male	-	-	+	-	-	-	-	-	E4V5M6	174	80	86	18	97	38.2	0	E4V5M6	165	82	80	16	95	37.9	+	-	-	-	-	-	-	Unknown	22.3	10.9	Levofloxacin (po)	5	None	-	-	-
22	67	Male	+	-	-	-	-	-	-	-	E4V5M6	120	80	70	16	96	36.5	0	E4V5M6	123	87	72	18	96	36.5	-	-	+	+	-	-	-	Unknown	8.4	7.47	None	0	High care unit	-	-	-

The youngest patient was 32 years old, while the oldest was 91 years old (median: 65.5 (IQR: 49.3-63.3) years). The most common chief complaints and findings in the ED were fever (18/22, 81.8%), chills (10/22, 45.5%), and localized pain or tenderness at the site of infection (8/22, 36.4%). In addition, patients often presented with a history of recent digestive symptoms, such as diarrhea, nausea/vomiting, or abdominal pain (6/22, 27.3%). Diabetes mellitus was reported in seven of the 22 (31.8%) patients, while hematological malignancies or solid tumors were reported in five of the 22 (22.7%) patients. In addition, four of the 22 (18.2%) patients had recent chemotherapy, and another four of the 22 (18.2%) patients presented with chronic kidney disease. Three (13.6%) patients had no comorbidities.

Patients with recent chemotherapy were more likely to be admitted at initial presentation to the ED compared with those who were discharged (4/11 (18.2%) versus 0/11 (0%)). Furthermore, the C-reactive protein levels were higher in the admitted patients than in the discharged patients (median: 10.7 (IQR: 1.59-12.9) versus 3.41 (IQR: 1.49-10.8) mg/L).

Cellulitis and vertebral osteomyelitis (3/22, 13.6%, for both) were the most common sources of bacteremia, followed by an infected aortic aneurysm, colitis, and infected cysts (renal cyst and pancreatic cyst with concomitant empyema) (2/22, 9.1%, for all). Meanwhile, the sources of infections were not identified in nine patients (40.9%). The overall prognosis of *H. cinaedi *bacteremia was good; only one patient required surgical intervention, and none died in the hospital. Among the 11 discharged patients at initial presentation to the ED, seven (63.6%) completed their treatment as outpatients without any sequelae. Antibiotics were prescribed to these initially discharged patients only after the blood cultures turned positive.

*H. cinaedi* bacteremia and microbiological features

*H. cinaedi *strains were detected in aerobic bottles, and the incubation time ranged from 4 to 11 days (median: 5 (IQR, 5-7) days). Blood cultures yielded a positive result within five days of sampling in 11 of 20 patients (55%) and longer than five days in nine of 20 patients (45%). Two patients had missing data on the incubation time.

All *H. cinaedi* isolates were susceptible to carbapenems (meropenem and imipenem), amikacin, gentamicin, and minocycline. Fewer *H. cinaedi* isolates were susceptible to cefotiam (5/18, 27.8%), ceftriaxone (4/10, 40.0%), ciprofloxacin (0/10, 0.0%), and levofloxacin (3/17, 17.6%) (Table 3).

**Table 3:** Antimicrobial susceptibility of H. cinaedi isolates obtained from patients presenting to the emergency department We demonstrated antibiotics and a number of *H. cinaedi* samples susceptible/total number of the tested samples (%).

Antibiotics	Susceptibility of *H. cinaedi* (%)
Penicillin	
- Ampicillin	16/18 (88.9)
Cephalosporins	
- Cefazolin (first generation)	15/17 (88.2)
- Cefotiam (second generation)	5/18 (27.8)
- Ceftriaxone (third generation)	4/10 (40.0)
- Cefepime (fourth generation)	17/17 (100)
Tetracyclines	
- Minocycline	17/17 (100)
Carbapenems	
- Meropenem	10/10 (100)
Fluoroquinolones	
- Levofloxacin	3/17 (17.6)
- Ciprofloxacin	0/10 (0.0)
Aminoglycosides	
- Gentamicin	17/17 (100)
- Amikacin	17/17 (100)
Sulfonamides	
- Trimethoprim-sulfamethoxazole	9/10 (90.0)

Management of *H. cinaedi* bacteremia

Patients with *H. cinaedi *bacteremia were treated with antibiotics administered through different routes (Table 4).

**Table 4:** Intravenous and oral antibiotics for patients with H. cinaedi bacteremia

Intravenous antibiotics chosen as definitive therapy (n=15 patients)	
- Minocycline, n (%)	5 (33.3)
- Cefazolin (first-generation cephalosporins)	3 (20.0)
- Sulbactam/ampicillin	3 (20.0)
- Ampicillin	1 (6.7)
- Tazobactam/piperacillin	1 (6.7)
- Cefotiam (second-generation cephalosporin)	1 (6.7)
- Cefepime (fourth-generation cephalosporin)	1 (6.7)
Oral antibiotics chosen as definitive therapy (n=20 patients)	
- Minocycline, n (%)	9 (45.0)
- Levofloxacin	3 (15.0)
- Doxycycline	3 (15.0)
- Ampicillin	2 (10.0)
- Cefaclor (first-generation cephalosporin)	2 (10.0)
- Cefditoren pivoxil (third-generation cephalosporin)	1 (5.0)

Of them, one patient (1/21, 4.8%) was treated with intravenous antibiotics alone, 14 (14/21, 66.7%) patients were treated with intravenous antibiotics and subsequently oral antibiotics, and six patients (6/21, 28.6%) were treated with oral antibiotics alone. The median total duration of the antimicrobial treatment was 20.5 days (IQR: 14.0-29.3). The duration of the antibiotic treatment exceeded 40 days in six patients with complex infections: vertebral osteomyelitis (three), infected aortic aneurysms (two), and cyst infection (one).

The frequently administered intravenous antibiotics were minocycline (5/15, 33.3%), cefazolin (a first-generation cephalosporin), and sulbactam/ampicillin (3/15, 20.0% for both). Minocycline was the most common oral antibiotic (9/20 45.0%), followed by levofloxacin and doxycycline (3/20, 15.0% for both).

Only one patient required a surgical stent graft replacement for an aortic aneurysm infected with *H. cinaedi*. Of note, one patient recovered from the infection without receiving antibiotic treatment. The patient was hospitalized and treated for hypokalemia and discharged with potassium correction alone.

## Discussion

In this descriptive study conducted at a single ED in Japan, we reported the patient characteristics and common clinical practices for *H. cinaedi* infections. The patients with *H. cinaedi* infections presented to the ED with vague clinical features, making it difficult to obtain an accurate diagnosis, and complicated infections were often the cases, including vertebral osteomyelitis and infected aortic aneurysms, unlike the previously reported cases in the primary care settings. Of note, *H. cinaedi* was detected only in blood cultures, and the duration of blood cultures exceeded five days in 45% of the patients.

Our study adds to the existing literature by providing a more detailed picture of *H. cinaedi *infections in emergency settings. In this study, patients with complicated cases, such as vertebral osteomyelitis, infected aortic aneurysms, and infected cysts, were more commonly reported in emergency settings; these cases were primarily documented in case reports conducted in primary care settings [14-21]. These complicated cases required antibiotic therapy for over 40 days; however, the prognoses were not poor with appropriate treatments, with only one patient requiring surgical intervention. Meanwhile, stable patients with *H. cinaedi* bacteremia can be safely managed in outpatient settings with close follow-ups, provided that they do not have any complicated concomitant source of infections and are not immunocompromised. Indeed, the clinical courses and outcomes, such as complications and need for surgical treatments, in-hospital days, and mortality, were not aggravated in the present study. On the contrary, immunocompromised patients, especially those with recent chemotherapy, were likely admitted at the first visit to the ED in the present study, and previous studies also pointed out that those patients are at risk for recurrence [8,9,22-27].

The diagnoses of *H. cinaedi* would have been missed when the general observation period for blood cultures was employed, as the time to positive blood culture was longer than five days. Our bacteriological laboratory employed a minimum observation period of seven days for blood cultures, which potentially facilitated the detection of *H. cinaedi* bacteremia in a greater number of patients. Furthermore, *H. cinaedi* was detected only in blood cultures and not in other specimens, such as bone and vessel wall tissues. These findings highlight the importance of blood cultures for detecting *H. cinaedi* infections in EDs.

As regard the antibiotic of choice, the optimal choice has not yet been known. Although there has not been sufficient evidence and recommended guidelines, susceptibility for tetracycline, aminoglycosides, and carbapenem and resistance to quinolones and macrolides have been reported [1]. We primarily treated with intravenous or oral tetracycline for *H. cinaedi *infections, and indeed all *H. cinaedi *isolates were susceptible to tetracyclines. Furthermore, the treatment of complex infections often necessitates a prolonged course of antimicrobial therapy to achieve a complete cure. Indeed, the present study demonstrated that approximately 30% of the patients underwent antimicrobial therapy for more than 40 days and were diagnosed with complex infections: three patients had vertebral osteomyelitis, two had infected aortic aneurysms, and one had a cyst infection. We also recommend a 14-day antibiotic course for most patients, with a longer duration considered for immunocompromised patients to prevent recurrence [10]. The optimum duration of treatment might vary depending on the specific source of infection in each patient.

This study had several limitations. First, it was descriptive in nature, and no control groups were established for comparison; hence, we could not draw conclusive clinical recommendations. Further studies are warranted to evaluate the optimal choice and route of antibiotic treatment for *H. cinaedi* infections. Second, the present study was performed at a single ED, which may have introduced a selection bias. The patients included in the present study might have more severe cases, as the study setting was a tertiary care centre. Finally, it is difficult to generalize the findings of the present study to other communities, where medical systems and resources differ. For example, admission criteria may vary in other countries, as well as the antibiotic preference and availability. Further studies of diverse patient populations are warranted.

## Conclusions

The diagnosis of *H. cinaedi* infections may be challenging owing to its rarity and unfamiliarity; performing blood cultures appropriately is the key to prompt and accurate diagnoses. In emergency settings, complicated cases of* H. cinaedi *bacteremia were more frequently observed. However, the prognosis of* H. cinaedi *bacteremia was good when it was managed with appropriate antibiotics, although the optimal treatment strategy has not yet been proven. Further studies are required to develop an individualized approach based on the patient’s infection and immunological status.
